# Influence of near-fault ground motions’ characteristics on the control performance of tuned viscous mass damper systems

**DOI:** 10.1371/journal.pone.0322535

**Published:** 2025-06-03

**Authors:** Lili Zhang, Zongcheng Liu, Jinhui Shi

**Affiliations:** School of Civil Engineering, Lanzhou University of Technology, Lanzhou, Gansu, China; Universiti Teknologi Malaysia, MALAYSIA

## Abstract

Near-fault ground motions, characterized by pronounced pulse and forward-directivity effects, present significant challenges to dampers’ effective performance in controlling seismic activity. This study provides an in-depth analysis of the influence of near-fault pulse-type ground motions’ characteristics on the concentration of peak responses in multi-story steel frame structures, the distribution patterns of weak layer locations, and Tuned Viscous Mass Dampers’ (TVMDs) effective control. The results indicate that near-fault ground motions’ pulse effect, forward-directivity effect, and spectral coefficients affect the distribution of maximum inter-story drift ratios along the building height significantly. Notably, the forward-directivity effect amplifies structural responses and diminishes TVMDs control’s effectiveness. In addition, ground motions with smaller spectral coefficients lead to larger inter-story drift responses. The effectiveness of TVMDs’ “damping enhancement” effect is determined jointly by their mass ratio and the pulse period of the ground motion records. This study provides important theoretical foundation for the seismic design of multi-story steel frame structures and the earthquake-reduction design using TVMDs under near-fault ground motions.

## 1. Introduction

Currently, in the field of structural vibration control technology, passive control methods that use Tuned Mass Dampers (TMD) are applied widely by incorporating tuning devices within structures [1–7]. Traditional vibration control methods with TMDs demonstrate optimal performance as the tuning mass increases. However, practical applications are often limited by the tuning mass available. To address this issue, Saito et al. [8] and Ikago et al. [9,10] proposed Tuned Viscous Mass Dampers (TVMDs). This device comprises one elastic element, one damping element, and a rotating mass element constructed from a flywheel, which Smith et al. [11] defined later as “inerter”. The inertial mass element is connected to the flywheel with materials such as gear racks or screw ball bearings, and provides an inertial control force that is proportional to the relative acceleration at both ends. This proportionality coefficient is referred to as the characterization mass (or inertial coefficient). Through mechanical design, the inertial element’s characterization mass can reach over a thousand times its physical mass [12].

The principal trends in the research on TVMD can be summarized into four categories: (1) parameter optimization; (2) performance simulation; (3) combination of TVMD with other dampers, and (4) practical engineering applications. Optimizing TVMD’s parameters is the first step in ensuring their superior performance. Various classical optimization techniques, including fixed-point theory (*H*_∞_ optimization) [9], *H*_2_ optimization [13,14], acceleration response control optimization [15], dual control optimization for displacement and acceleration responses [16], effective damping ratio optimization [17], and energy dissipation maximization [18], have led to closed-form solutions for the optimal frequency ratio and damping ratio. Further, several numerical optimization programs that consider different optimization objectives have been proposed [19–21]. Numerical studies have been conducted on TVMD’s vibration control performance in both linear and nonlinear structural systems [10,22–26]. Other researchers have focused on novel tuned mass dampers that replace traditional damping elements with TVMD [27–31]. Moreover, cross-layer, multi-modal, and nonlinear TVMD have also garnered attention [32–34]. With respect to practical engineering applications, the first civil engineering structure in the world that used an inertial damping system was the Sendai NTT building [35].

There are various types of pulse excitations in practical applications, such as near-fault ground motions [36–39] and transient gusts [40–42]. Compared to stationary excitations, near-fault pulse-type ground motions, characterized by short duration and high energy input, produce stronger dynamic responses and are more likely to cause severe structural damage. Therefore, controlling the peak responses under pulse excitations is one of the most important current challenges. Some researchers have investigated tuned dampers’ seismic control performance under the influence of near-fault pulse-type ground motions in response to excessive vibration [43–52]. Although the use of tuned dampers to control structural seismic responses is no longer an innovative application, there is currently a lack of research on TVMD’s effectiveness in controlling structural responses specifically under near-fault pulse-type ground motions. Moreover, there is a particular scarcity of investigations into the correlation between TVMD’s performance and the characteristics of near-fault pulse-type ground motions. Specifically, there is a significant gap in understanding the impact of characteristics such as pulse period, forward-directivity effects, and spectral coefficients PGA/PGV (Peak Ground Acceleration, PGA; Peak Ground Velocity, PGV), on the damping control performance of TVMDs. Thus, the effectiveness of the “damping enhancement” that TVMD provide under such conditions warrants further systematic study. In addition, research on near-fault pulse-type ground motion characteristics’ effect on the concentration of responses and the distribution of weak layer locations (the floors in a multi-story steel frame structures where the maximum inter-story drift ratio occurs under near-fault ground motions) remains insufficient.

This study selected 288 near-fault ground motion records as excitation inputs and uses OpenSees to conduct dynamic time history analyses on a typical multi-story steel frame structures equipped with TVMD, Viscous Dampers (VDs), and no damping devices. The analysis investigates the effects of pulse period, forward-directivity effects, and spectral coefficients PGA/PGV on the concentration of peak structural responses and the distribution patterns of weak layer locations along the building height. Based upon the “damping enhancement” effect, the damping control’s sensitivity to ground motion characteristics (such as pulse period, forward-directivity effects, and spectral coefficients PGA/PGV), damper types, and mass ratios, and its effectiveness is examined. By comparing TVMD and VD’s responses, their efficiency in absorbing and dissipating seismic energy under different mass ratios and seismic conditions, this study explores the relation between TVMD’s “damping enhancement” effect and structures’ nonlinear development. In addition, the influence of pulse period, forward-directivity effects, and the spectral coefficients, PGA/PGV, on the effectiveness of the “damping enhancement” that TVMD provide is analyzed and discussed.

## 2. Characteristics of near-fault ground motions

Near-fault ground motions exhibit more complex seismic characteristics than far-field ground motions, and primarily include the following: velocity pulse effect; directivity effect; hanging wall effect; fling-step effect, and concentration of near-field ground motions [53–55].

The velocity pulse effect arises from two primary causes: first, the directivity effect associated with the rupture, which manifests as bidirectional velocity pulses that occur predominantly in components perpendicular to the fault direction. Notably, pulses recorded during the forward directivity effect are more pronounced than those recorded during the backward directivity effect. Second, the effect can also be attributed to permanent ground displacement, which results in unidirectional velocity pulses observed primarily in components parallel to the direction of the fault slip [56]. When a fault ruptures, the seismic waves generated by the sub-sources propagate toward the rupture front at approximately shear wave velocities, which causes their energy to arrive at the fault zone simultaneously within a short time. This rapid accumulation of energy leads to a high-amplitude and short-duration pulse effect, referred to as the “forward-directivity effect [57].” The ratio of PGA/PGV reflects the ground motion’s dominant frequency and energy components, and serves as a measure of the seismic signal’s spectral characteristics. Seismic motions characterized by intense acceleration and longer durations typically exhibit lower PGA/PGV values, as observed in near-fault pulse-type ground motions rich in low-frequency components. Conversely, ground motions with higher frequencies and shorter durations tend to have larger PGA/PGV values.

## 3. Design and modeling of structures with tvmd damping systems

According to reference [23], the motion equations of the shear-type multi-degrees-of-freedom system with an attached TVMD under seismic acceleration excitation can be expressed by Eq. (1):


$$\text{M}\ddot{\text{x}}+\text{C}\dot{\text{x}}+\text{Kx}=-\text{Mr}{\ddot{\text{x}}}_{0}$$ (1)

In the equation, $x={\left[x_{\text{s}},x_{\text{d}}\right]}^{\text{T}}$ is the displacement response $x_{\text{s}}$ of the seismic-resistant structure and the relative displacement $x_{\text{d}}$ of the attached TVMD; $\ddot{𝐱}$ and $\dot{x}$ are the acceleration and velocity response of the structure, respectively; $r={\left[1,\cdots ,1,0,\cdots ,0\right]}^{\text{T}}$ is the ground motion identification vector, and M, C, and K are the mass, damping, and stiffness matrices of the system, respectively.


$$M=\left[\begin{array}{cc} M_{s} & 0\\ 0 & M_{d} \end{array}\right],C=\left[\begin{array}{cc} C_{s} & 0\\ 0 & C_{d} \end{array}\right],K=\left[\begin{array}{cc} K_{s}+K_{d11} & K_{d12}\\ K_{d21} & K_{d22} \end{array}\right]$$ (2)

In the equation, $M_{s}$, $K_{s}$, and $C_{s}$ are the mass, damping, and stiffness matrices of the structure, respectively; $M_{d}=diag\left\{m_{\text{d}}\right\}$, $C_{d}=diag\left\{c_{\text{d}}\right\}$, and $K_{d22}=diag\left\{k_{\text{d}}\right\}$ are the mass, damping and stiffness matrices of the TVMD, respectively. The construction of the additional stiffness matrices $K_{d11}$, $K_{d12}$, and $K_{d21}$ is the same as in reference [23]. The deformation of the TVMD can be approximated by the inter-story displacement difference. Therefore, in the preliminary design, the mode-shaped vector was obtained by performing a mode-shaped analysis of the seismic structure. The mode-shaped mass ${\hat{m}}_{\text{d},ji}$ corresponding to the *j*^th^ mode shape with the installed TVMD is estimated using Eq. (3).


$$m_{\text{d},i}{\left(\phi_{j,i}-\phi_{j,i-1}\right)}^{2}={\hat{m}}_{\text{d},ji}$$ (3)

In the equation, $\phi_{j,i}$ and $\phi_{j,i-1}$ are the *i*^th^ and *(i-1)*^th^ elements of the mode shape vector $\Phi_{j}$, respectively. Then, the modal mass ratio ${\hat{\mu }}_{\text{d},ji}$ of the TVMD is given by


$${\hat{\mu }}_{\text{d},ji}={\hat{m}}_{\text{d},ji}/\left(\Phi_{j}^{T}M_{s}\Phi_{j}\right)\;$$ (4)

Combining Eqs. (3) and (4), the modal mass ratio ${\hat{\mu }}_{\text{d},ji}$ of the TVMD can be used to obtain the apparent mass $m_{\text{d},i}$ of the TVMD. The design stiffness $k_{\text{d},i}$ and design damping coefficient $c_{\text{d},i}$ of the TVMD can be obtained using Eqs. (5) and (6), respectively.


$$k_{\text{d},i}{=}^{\text{opt}}\beta_{\text{d},j}^{2}\omega_{j}^{2}m_{\text{d},i}$$ (5)

$$c_{\text{d},i}=2^{\text{opt}}\zeta_{\text{d}}^{,j\text{opt}}\beta_{\text{d},j}\omega_{j}m_{\text{d},i}$$ (6)

In the equation, βoptd,j and ζoptd,j are the optimal frequency and damping ratio of the TVMD, respectively. Based on the fixed-point theory, numerous researchers have proposed various design formulas. In this paper, the parameters were calculated using the results proposed by Ikago et al. [23].


βoptd,j=11−μd,ζoptd,j=μd23μd(1−μd)(2−μd) (7)

**Fig 1:**
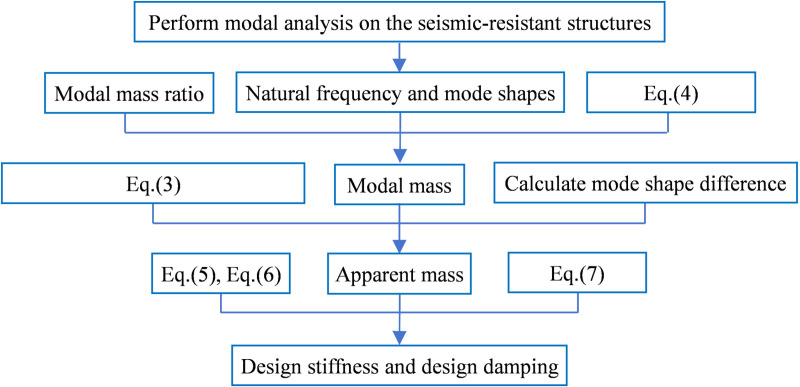
The Flowchart of TVMD Parameter Design Process.

The specific parameter design process is shown in Fig 1.

## 4. Influence of ground motion characteristics on the distribution patterns of maximum responses in seismic structures

**Fig 2:**
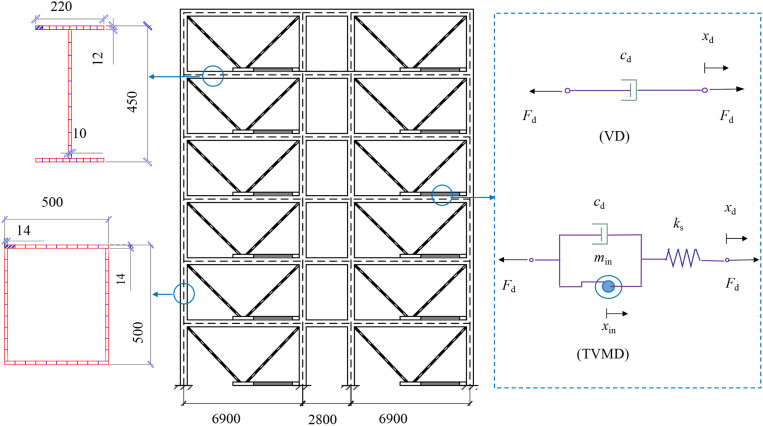
Schematic diagram of seismic and energy dissipated structures (Unit: mm).

This study focuses on a comprehensive teaching building at a vocational school, which consists of six stories above ground, each with a height of 3.6 meters. One of the transverse frames is selected as the computational unit, as illustrated in Fig 2. Modeling and analysis are performed using OpenSees finite element software. Detailed information about the structure and model can be found in reference [47]. The relation between the additional damping ratios of VD and TVMD is established as follows: $\xi_{\text{d}}^{\text{TVMD}}=\xi_{\text{d}}^{\text{VD}}$. To illustrate the mass ratio’s effect on TVMD’s damping performance visually, the VD’s additional damping ratio is expressed in terms of the mass ratio that corresponds to the same additional damping ratio of the TVMD.

Following the recommendations of Connor et al. [56], peak-to-peak velocity (PPV) pulse identification is performed on near-fault ground motions to calculate the normalized cumulative squared velocity (NCSV). The differences in NCSV are then used to determine whether the near-fault ground motion records exhibit significant pulsing characteristics. Based on the aforementioned criteria for selecting ground motion records, 288 near-fault pulse-type ground motion records (S1 Table) are obtained from the Pacific Earthquake Engineering Research Center (PEER), with detailed information provided in reference [56].

### 4.1. Influence of pulse period on the distribution patterns of maximum responses in seismic structures

**Fig 3:**
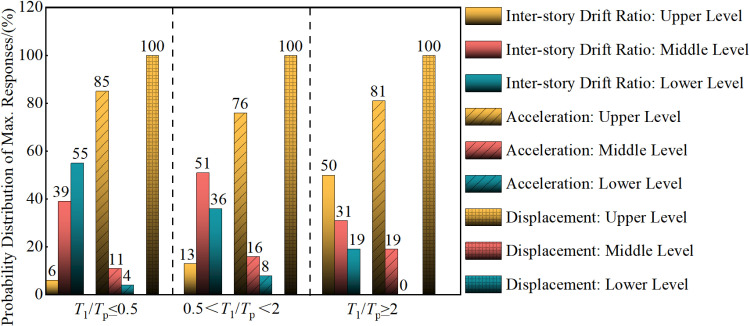
Distribution probability of maximum responses in seismic structures.

The pulse period, $T_{\text{p}}$, is a key parameter of near-fault pulse-type ground motions that influences maximum structural responses’ distribution patterns significantly. Based upon the different ratios of $T_{1}/T_{\text{p}}\;$ (in which $T_{1}$ is the structure’s fundamental period and $T_{\text{p}}$ is the ground motion records’ pulse period), the 288 near-fault pulse-type ground motion records are divided into three groups: $T_{1}/T_{\text{p}}\;\leq 0.5$ (a total of 164 records), $0.5<T_{1}/T_{\text{p}}\;<2$ (a total of 108 records), and $T_{1}/T_{\text{p}}\;\geq 2$ (a total of 16 records) [58]. The pulse period, $T_{\text{p}}$ ’s, influence on the distribution patterns of maximum responses in seismic structures under each group of near-fault ground motion records is discussed. Under the design seismic conditions (PGA = 0.30g), the distribution patterns of maximum inter-story drift ratios, maximum accelerations, and maximum displacements along the building height are illustrated in Fig 3.

As illustrated in Fig 3, the vertical axis represents the probability ($P=\frac{n}{N}$, where n is the total number of ground motion records in which the maximum response occurs at the target location, and N is the total number of ground motion records in each category), which indicates the likelihood that the maximum response (inter-story drift ratio, acceleration, and displacement) occurs at the upper, middle, or lower levels. The maximum displacement in seismic structures occurs consistently at the top level, indicating that the ground motion’s pulse period does not influence the probability distribution of maximum displacement along the building height. Instead, the probability distribution is as follows: when $T_{1}/T_{\text{p}}\;\leq 0.5$, the probabilities of occurrence in the upper (5th-6th floors), middle (3rd-4th floors), and lower (1st-2nd floors) levels are 85%, 11%, and 4%, respectively; when $0.5<T_{1}/T_{\text{p}}\;<2$, these probabilities are 76%, 16%, and 8%, and when $T_{1}/T_{\text{p}}\;\geq 2$, the probabilities are 81%, 19%, and 0%. This demonstrates that maximum acceleration is highly likely to occur in the structure’s upper levels. This phenomenon derives from the interaction of several factors, including vibration modes, mass distribution, seismic wave propagation characteristics, structural stiffness distribution, and dynamic coupling effects. In this study, the seismic structure’s stiffness decreases progressively from the bottom to the top and results in increased flexibility in the upper structure, which leads to larger acceleration responses.

The probability distribution of maximum inter-story drift ratios in seismic structures along the building height is as follows: when $T_{1}/T_{\text{p}}\;\leq 0.5$, the probabilities of occurrence in the upper, middle, and lower levels are 6%, 39%, and 55%, respectively; when $0.5<T_{1}/T_{\text{p}}\;<2$, these probabilities are 13%, 51%, and 36%, and when $T_{1}/T_{\text{p}}\;\geq 2$, the probabilities are 50%, 31%, and 19%.

**Fig 4:**
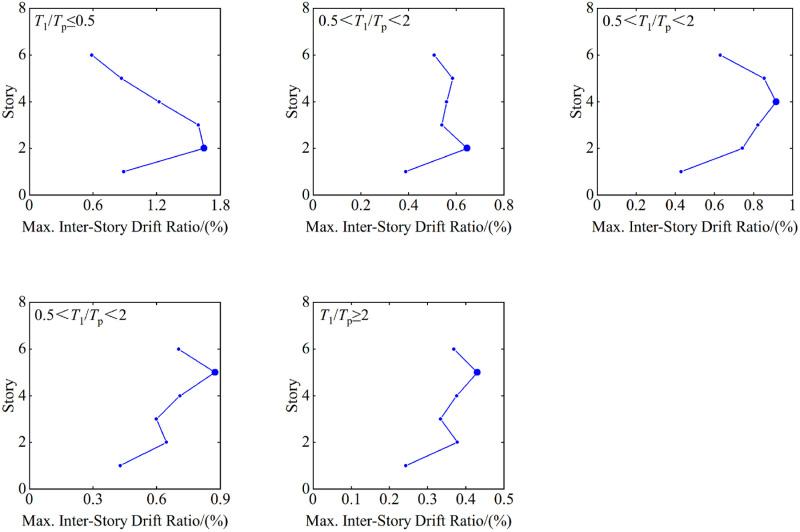
Influence of pulse period on the distribution of maximum inter-story drift ratios.

The maximum inter-story drift ratio responses for each group of pulse period near-fault ground motion records are illustrated in Fig 4.

As illustrated in Fig 4, the pulse period, $T_{\text{p}}$, of near-fault pulse-type ground motion records in seismic structures influences the distribution patterns of maximum inter-story drift ratios ($\theta =\frac{\Delta u_{i}}{h_{i}}$, where $\Delta u_{i}=u_{i}-u_{i-1}$ is the horizontal displacement of the *i*^th^ floor, defined as the displacement difference between the *i*^th^ and (*i*-1)^th^ floors at corresponding locations in the floor plan; $h_{i}$ is the story height of the *i*^th^ floor) along the building height significantly. When $T_{1}/T_{\text{p}}\;\leq 0.5$, $T_{\text{p}}$ is long relative to the structure’s fundamental period and $T_{\text{1}}$, the pulse-type ground motion’s effects, are concentrated in lower frequency responses, and lead to maximum inter-story drift ratios concentrated primarily in the lower levels of the structure. Because of the longer pulse period, the inter-story drift ratios in the upper levels are relatively small, while those in the lower levels increase significantly, indicating that the pulse-type ground motion has a more pronounced effect on the lower structure, and could potentially lead to damage.

When $0.5<T_{1}/T_{\text{p}}\;<2$, $T_{\text{p}}$ is the near fundamental period, $T_{\text{1}}$, of the structure, the ground motion’s pulse effects resonate with the structure’s natural frequency, which allows maximum inter-story drift ratios to occur potentially across the entire height of the structure with a relatively uniform distribution. The resonance effect results in larger inter-story drift ratios at all levels, particularly in the middle and upper sections. This increased response of the structure overall may lead to substantial damage or noticeable plastic deformations.

When $T_{1}/T_{\text{p}}\;\geq 2$ ,$,T_{\text{p}}$is short relative to the fundamental period, $T_{\text{1}}$, the pulse-type ground motion’s effects cluster in higher frequency responses, which results in maximum inter-story drift ratios concentrated primarily in the structure’s upper levels. Because of the shorter pulse period, the inter-story drift ratios in the lower levels remain relatively small, while those in the upper levels increase significantly, indicating that the pulse-type ground motion has a more pronounced effect on the upper structure, which potentially could cause damage in the upper portions.

In summary, the pulse period influences the distribution patterns of maximum inter-story drift ratios in seismic structures significantly, particularly when the pulse period approaches the structure’s natural period, as this condition is prone to induce resonance effects that increase the structural response substantially. Therefore, in structural design and protective measures, it is essential to consider the pulse period’s effect adequately and to employ various technical methods to control inter-story drift ratios effectively, which will enhance the structure’s seismic performance thereby.

### 4.2. Influence of forward-directivity effect on the distribution patterns of maximum responses in seismic structures

**Fig 5:**
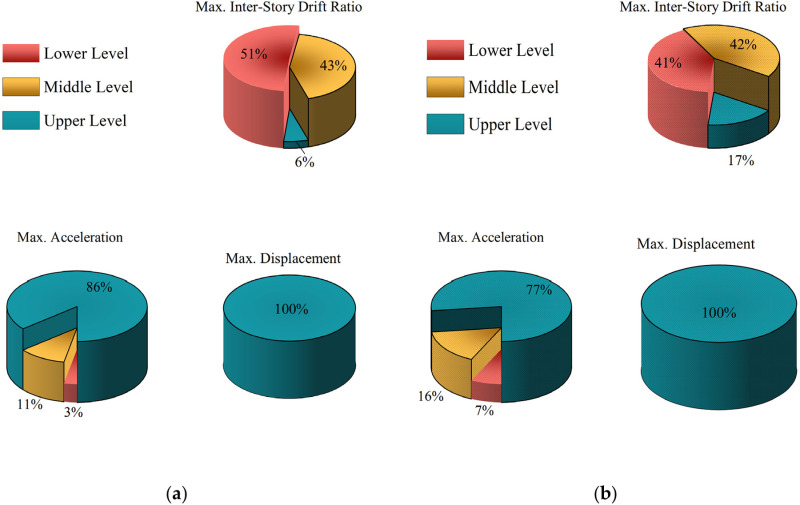
Influence of forward-directivity effect on the distribution of maximum responses in seismic structures: (a) with forward-directivity effect; (b) without forward-directivity effect.

The statistical analysis indicates that there are 150 records of near-fault ground motions that exhibit forward-directivity effects and 138 records that do not. The influence of these two categories of near-fault ground motion records on the distribution patterns of maximum responses in seismic structures is discussed. Under the design seismic conditions (PGA = 0.30g), the distribution patterns of maximum inter-story drift ratios, maximum accelerations, and maximum displacements along the building height are illustrated in Fig 5.

In Fig 5, it can be observed that the maximum displacement occurs at the seismic structure’s top level, which indicates that the ground motion’s forward-directivity effect does not affect the distribution probability of maximum displacement along the height of the structure. The distribution probabilities of maximum acceleration along the structure are as follows: in the presence of forward-directivity effects, the probabilities of occurrence at the upper, middle, and lower levels are 86%, 11%, and 3%, respectively; without forward-directivity effects, the probabilities are 77%, 16%, and 7%, respectively. The distribution probabilities of maximum inter-story drift ratios are as follows: with forward-directivity effects, the probabilities at the upper, middle, and lower levels are 6%, 43%, and 51%, respectively, and without forward-directivity effects, they are 17%, 42%, and 41%.

This indicates that under ground motions that exhibit forward-directivity effects increase the likelihood that maximum inter-story drift ratios will occur in the lower levels of the structure, and necessitates greater attention to the lower portions of the structure. This phenomenon can be attributed to the lower levels’ relatively higher inter-story stiffness. During forward-directivity-induced ground motions, the concentrated release of energy may prevent the stiffer lower levels from dissipating the abruptly input energy effectively, and lead to the potential for plastic deformation in these lower regions. Such nonlinear behavior may result in a significant increase in inter-story drift ratios at the lower levels, making it more likely for maximum inter-story drift ratios to occur there compared to ground motions without forward-directivity effects.

**Fig 6:**
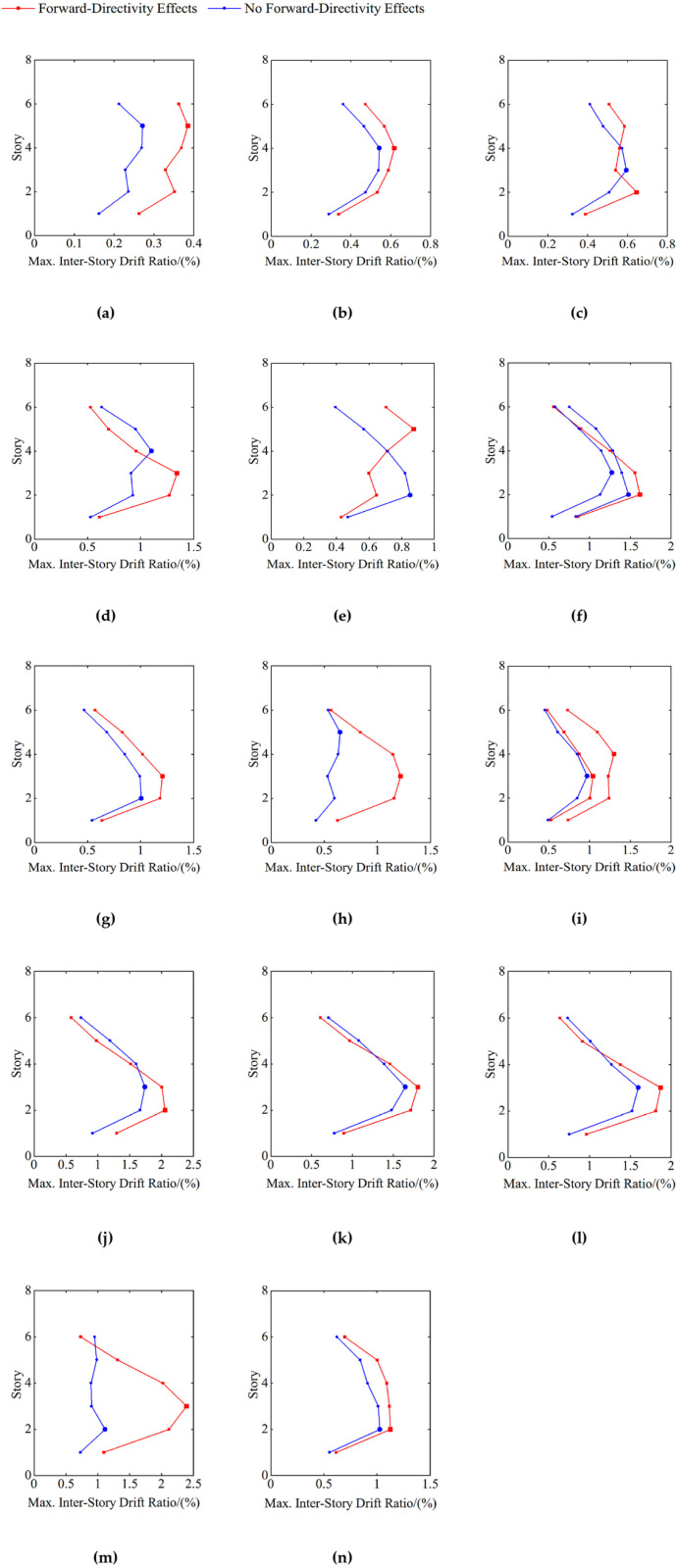
Directivity effects on the distribution of maximum inter-story drift ratios. (a) $T_{\text{p}}=0.6\text{s}$; (b) $T_{\text{p}}=0.7\text{s}$; (c) $T_{\text{p}}=0.8\text{s}$; (d) $T_{\text{p}}=0.9\text{s}$; (e) $T_{\text{p}}=1.0\text{s}$; (f) $T_{\text{p}}=1.1\text{s}$; (g) $T_{\text{p}}=1.4\text{s}$; (h) $T_{\text{p}}=1.6\text{s}$; (i) $T_{\text{p}}=2.5\text{s}$; (j) $T_{\text{p}}=2.9\text{s}$; (k) $T_{\text{p}}=4.9\text{s}$; (l) $T_{\text{p}}=5.4\text{s}$; (m) $T_{\text{p}}=6.7\text{s}$; (n) $T_{\text{p}}=7.5\text{s}$.

The near-fault pulse-type ground motions with the same pulse period and a PGA/PGV difference that does not exceed 10% are analyzed, and the seismic structure’s inter-story drift ratios under the design earthquake conditions (PGA = 0.30g) with and without forward-directivity effects are illustrated in Fig 6. In the figure, the larger red solid squares and blue solid circles represent the seismic structure’s maximum response values under the conditions of with and without forward-directivity effects, respectively.

To analyze forward-directivity effects’ influence on seismic structures’ inter-story drift ratio response, the forward-directivity effects influence coefficient, $f_{\text{FD}}^{\text{SS}}$, is defined based upon the maximum value of the inter-story drift ratio. The mathematical expression is as follows:


$$f_{\text{FD}}^{\text{SS}}=\frac{\theta_{\max}^{\text{FD}}}{\theta_{\max}}$$ (8)

In the equation, $f_{\text{FD}}^{\text{SS}}$ represents the forward-directivity effects influence coefficient, in which SS denotes the seismic structure and FD indicates the forward-directivity effects; $\theta_{\max}^{\text{FD}}$ is the structure’s maximum inter-story drift ratio under forward-directivity effects, and $\theta_{\max}$ is the structure’s maximum inter-story drift ratio under non-forward-directivity effects.

The statistical values of the forward-directivity effects influence coefficient are presented in Table 1.

**Table 1:** Influence coefficient of forward-directivity effects.

$T_{\text{p}}/(\text{s})$	$\theta_{\max}^{\text{FD}}/(\;$	$\theta_{\max}/(\;$	$f_{\text{FD}}^{\text{SS}}$
0.6	0.3856	0.2719	1.4182
0.7	0.6187	0.5434	1.1386
0.8	0.6464	0.5955	1.0856
0.9	1.3438	1.1023	1.2190
1.0	0.8751	0.8522	**1.0268** ^**b**^
1.1	1.6166	1.4791 (1.2744) ^a^	1.0930 (1.2685) ^a^
1.4	1.2061	1.0064	1.1984
1.6	1.2196	0.6489	1.8795
2.5	1.0414 (1.3022) ^a^	0.9722	1.0711 (1.3393) ^a^
2.9	2.0549	1.7403	1.1808
4.9	1.8034	1.6506	1.0926
5.4	1.8721	1.5967	1.1724
6.7	2.3914	1.1128	**2.1490** ^**b**^
7.5	1.1248	1.0267	1.0955

^a^The values (*) represent the second values of $\theta_{\max}^{\text{FD}}$, $\theta_{\max}$ and $f_{\text{FD}}^{\text{SS}}$.

^b^ The bold numbers indicate the maximum and minimum values of the influence coefficient of forward-directivity effects.

In Fig 6 and Table 1, it can be observed that the range of the forward-directivity effects influence coefficient is from 2.1490to 1.0268, with a mean of 1.2774. This indicates that the near-fault pulse-type ground motion records’ forward-directivity effects amplify seismic structures’ response significantly. This amplification is attributable to the high-amplitude velocity pulses that often accompany forward-directivity effects and result in a notable increase in the ground motion records’ PGA and PGV. Although the duration of these records is relatively short, the energy is concentrated and thus leads to pronounced pulse effects. Consequently, the inter-story drift ratio increases significantly, heightening the shear deformation and potential risk of damage to the structure.

### 4.3. Influence of PGA/PGV on the distribution pattern of maximum responses in seismic structures

For ease of description, the ratio of PGA to PGV is defined as the spectral coefficient, $f_{\text{g}}$, given by:


$$f_{\text{g}}=\frac{\text{PGA}}{\text{PGV}}$$ (9)

In the equation, $f_{\text{g}}$ represents the spectral coefficient; PGA denotes the peak ground acceleration, and PGV signifies the peak ground velocity.

**Fig 7:**
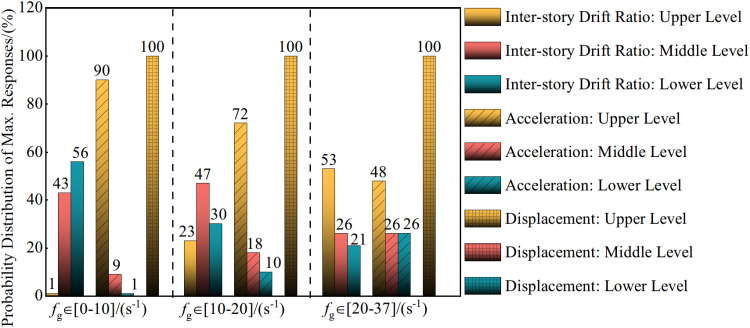
The influence of spectral characteristics on the distribution of maximum responses in seismic structures.

Statistical analysis of 288 near-fault pulse-type ground motion records reveals that the spectral coefficient, $f_{\text{g}}$, ranges from 1.376s^-1^ to 36.230s^-1^, which attempts to encompass the seismic spectral characteristics to the greatest extent possible. This facilitates a discussion of the seismic spectral characteristics’ influence on the distribution patterns of maximum responses in seismic structures. Under the design earthquake condition (PGA = 0.30g), the distribution patterns of maximum inter-story drift ratios, maximum accelerations, and maximum displacements along the height of the structure are illustrated in Fig 7.

Fig 7 shows that the maximum displacement occurs at the seismic structure’s top level, which demonstrates that the spectral coefficient, $f_{\text{g}}$ does not influence the distribution probability of maximum displacement along the floors. Instead, the distribution probabilities of maximum acceleration along the floors are as follows: when $\begin{array}{c} f_{\text{g}}\in \text{[0},{\text{10]s}}^{\text{-1}} \end{array}$ the probabilities for the upper, middle, and lower levels are 90%, 9%, and 1%, respectively; when $\begin{array}{c} f_{\text{g}}\in \text{[10},{\text{20]s}}^{\text{-1}} \end{array}$ the probabilities are 72%, 18%, and 10%, and when $\begin{array}{c} f_{\text{g}}\in \text{[20},{\text{37]s}}^{\text{-1}} \end{array}$ the probabilities are 48%, 26%, and 26%. This indicates that $f_{\text{g}}$ affects the distribution of maximum acceleration responses in the structure significantly. As $f_{\text{g}}$ increases, the location of the maximum acceleration response shifts downward gradually along the structure’s height.

The distribution probabilities of maximum inter-story drift ratios along the seismic structure’s floors are as follows: when $\begin{array}{c} f_{\text{g}}\in \text{[0},{\text{10]s}}^{\text{-1}} \end{array}$, the probabilities for the upper, middle, and lower levels are 1%, 43%, and 56%, respectively; when $\begin{array}{c} f_{\text{g}}\in \text{[10},{\text{20]s}}^{\text{-1}} \end{array}$, the probabilities are 23%, 47%, and 30%, and when $\begin{array}{c} f_{\text{g}}\in \text{[20},{\text{37]s}}^{\text{-1}} \end{array}$ the probabilities are 53%, 26%, and 21%. This indicates that $f_{\text{g}}$ has a significant effect on the distribution of maximum inter-story drift ratio responses in the structure.

When $\begin{array}{c} f_{\text{g}}\in \text{[0},{\text{10]s}}^{\text{-1}} \end{array}$, the low-frequency components of the ground motion dominate, suggesting lower peak ground acceleration, but larger variations in velocity. Consequently, the ground motion’s influence on the structure is concentrated in the lower-frequency responses, and maximum inter-story drift ratios occur primarily in the structure’s lower levels. The upper levels exhibit relatively small inter-story drift ratios, while the lower levels experience a significant increase, which may lead to potential damage in the lower structure.

When $\begin{array}{c} f_{\text{g}}\in \text{[10},{\text{20]s}}^{\text{-1}} \end{array}$, maximum inter-story drift ratios can occur across the entire height of the structure, leading to a more uniform distribution. The inter-story drift ratios of all levels are relatively large, particularly in the middle and upper sections. This results in a greater structural response overall, which potentially could lead to significant structural damage or pronounced plastic deformation.

When $\begin{array}{c} f_{\text{g}}\in \text{[20},{\text{37]s}}^{\text{-1}} \end{array}$, the ground motion’s high-frequency components prevail, indicating higher peak ground acceleration, but smaller variations in velocity. Thus, the ground motion’s influence focuses on higher-frequency responses, and maximum inter-story drift ratios are concentrated primarily in the structure’s upper levels. The inter-story drift ratios in the lower levels are relatively small, while those in the upper levels increase significantly, and can potentially cause damage to the upper structure.

**Fig 8:**
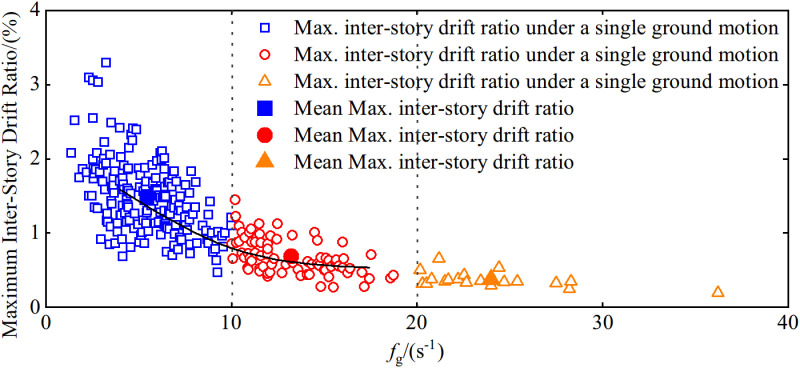
The influence of the spectral coefficient, $f_{\text{g}}$, on the maximum inter-story drift ratio response.

The maximum inter-story drift ratios and their averages for the seismic structure under the design seismic conditions (PGA = 0.30g) are presented in relation to different ranges of $f_{\text{g}}$, as shown in Fig 8.

As illustrated in Fig 8, during $f_{\text{g}}$ ’s transition from 1.38 to 36.23, the seismic structure’s maximum inter-story drift ratio decreases as the spectral coefficient increases. Ground motions with a higher $f_{\text{g}}$ contain more high-frequency components that result in rapid loading and unloading rates. Consequently, the inertial forces acting on the structure in the same direction do not persist for long, and lead to insufficient time for the responses at each level to accumulate. Thus, under ground motions with larger $f_{\text{g}}$, the building structure’s inter-story drift ratio responses are comparatively smaller. Conversely, ground motions with smaller $f_{\text{g}}$ distribute energy predominantly in the low-frequency range, which leads to slower loading and unloading rates. The sustained acceleration pulses in the same direction generate sufficient inertial forces that enable the structure to undergo greater deformations. Therefore, the responses are more significant. In summary, ground motions characterized by smaller $f_{\text{g}}$ are relatively more detrimental, as they can induce greater inter-story drift ratio responses in the building structure.

## 5. Ground motion characteristics’ influence on the control performance of tuned viscous mass dampers

### 5.1. Influence of pulse period on the control performance of TVMD

**Fig 9:**
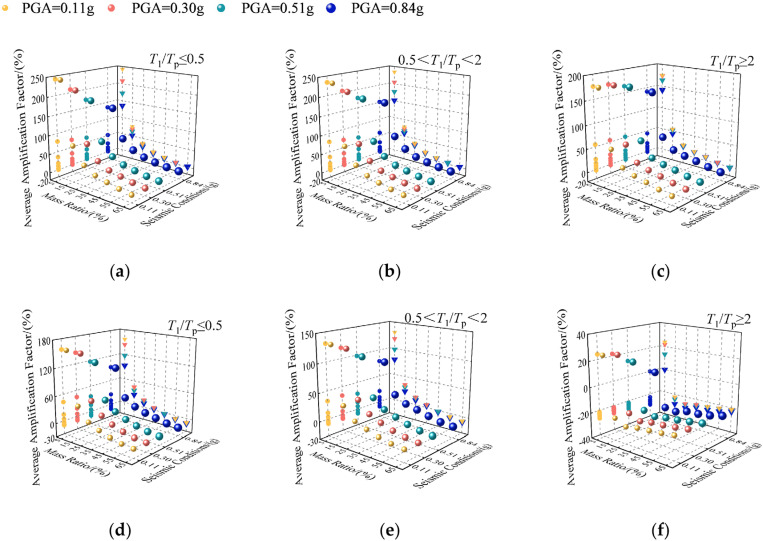
Relation between average amplification factors, mass ratio, and seismic conditions: (a) deformation; (b) deformation; (c) deformation; (d) damping force; (e) damping force; (f) damping force.

To validate the effectiveness of TVMD’s “damping enhancement” under different pulse period ground motions, TVMD’s average damping forces and deformation amplification factors compared to VD were analyzed statistically under various mass ratios ($\mu_{\text{d}}=0.05,0.15,0.25,0.35,0.45,0.55,0.65$) [59,60] and seismic conditions, including frequent (PGA = 0.11g), design (PGA = 0.30g), rare (PGA = 0.51g), and extremely rare (PGA = 0.84g) events, as shown in Fig 9.

As Fig 9 shows, under the same seismic conditions, as the mass ratio increases, the average amplification factors for deformation and damping force decrease gradually. Under a constant mass ratio, as the ground motion’s intensity increases, the average amplification factors show only slight changes. This indicates that the TVMD’s damping control performance does not diminish with increasing nonlinearity, and exhibits stable performance across multiple seismic conditions, including frequent, design, rare, and extremely rare events.

As illustrated in Figs 9(a), (b), and (c), when the mass ratio $\mu_{\text{d}}(0.55$, the average deformation amplification factor yields negative values. Figs 9(d), (e), and (f) demonstrate that when $T_{1}/T_{\text{p}}(2$ and $\mu_{\text{d}}(0.45$ as well as $T_{1}/T_{\text{p}}(2$ and $\mu_{\text{d}}(0.15$, the average damping force amplification factor also exhibits negative values. This indicates that under these circumstances, the TVMD cannot achieve the “damping enhancement” effect for the damping units.

In summary, when $T_{1}/T_{\text{p}}(2$ and $\mu_{\text{d}}(0.45$ as well as $T_{1}/T_{\text{p}}(2$ and $\mu_{\text{d}}(0.15$, the TVMD amplifies the damping force and deformation at the damper significantly, which allows it to dissipate more seismic energy and achieve more effective damping control, thereby reflecting the “damping enhancement” effect for the damping units. However, beyond these specific conditions, the TVMD fails to realize the “damping enhancement” effect for the damping units.

### 5.2. Influence of forward-directivity effect on TVMD’s control performance.

The standard deviation and range of the structural dynamic responses, including the maximum displacement, maximum acceleration, and maximum inter-story drift ratio, under near-fault ground motions are listed in Table 2.

**Table 2:** Standard Deviation and Range of Structural Dynamic Response.

Structure type	Standard deviation			Range		
	Maximum displacement	Maximum acceleration	Max inter-story drift ratio	Maximum displacement	Maximum acceleration	Max inter-story drift ratio
SRS	0.084	0.145	0.0061	0.426	0.614	0.0308
EDS-I	0.071	0.102	0.0051	0.359	0.370	0.0254
EDS-II	0.051	0.114	0.0036	0.275	0.393	0.0187

Note: the steel frame structure without dampers (referred to as the seismic-resistant structure, SRS), VD seismic-resistant structure (referred to as energy-dissipated structure I, EDS-I), and TVMD seismic-resistant structure (referred to as energy-dissipated structure II, EDS-II).

From Table 2, it can be observed that under the near-fault ground motions, the SRS, EDS-I, and EDS-II exhibited significant variability in the dynamic response. Therefore, judging the effectiveness of the VD and TVMD seismic control based solely on the structural dynamic response under a single ground motion would overlook the variability in both ground motions and structural responses. It is recommended that the mean earthquake-reduction coefficients from multiple ground motions be used to evaluate the damping control effects of the VD and TVMD on the peak response of the structure.

**Fig 10:**
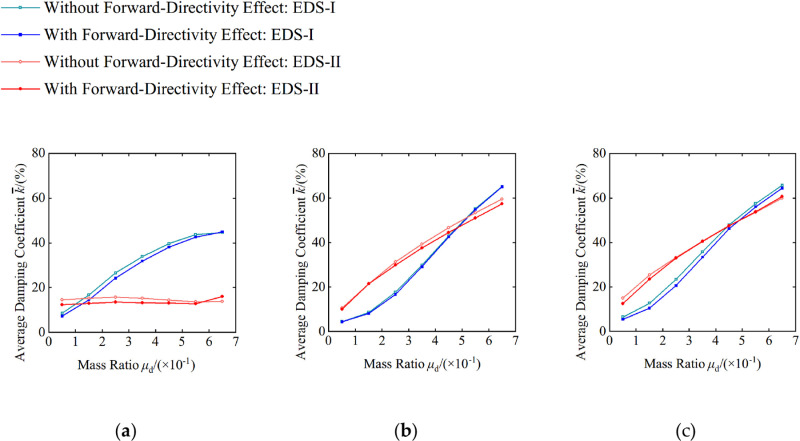
Forward-directivity effects’ influence on the average damping coefficient of maximum responses: (a) maximum acceleration; (b) maximum displacement; (c) maximum inter-story drift ratio.

Under the near-fault ground motion records with and without forward-directivity effects, the average seismic reduction coefficients for maximum acceleration, maximum displacement, and maximum inter-story drift ratio of Energy Dissipated Structure I (EDS-I, the structure with additional VD) and Energy Dissipated Structure II (EDS-II, the structure with additional TVMD) are shown in relation to mass ratio under the design earthquake conditions (PGA = 0.30g) in Fig 10.

Fig 10 illustrates that near-fault ground motion records’ forward-directivity effect influences the devices’ damping performance, and reduces the TVMD’s seismic control performance, in that displacement and acceleration are more pronounced than those of VD. Further, as the mass ratio increases, the reduction in the TVMD’s displacement control performance becomes more significant. Conversely, the weakening of TVMD’s control performance with respect to the inter-story drift ratio is less than that of VD, and this weakening diminishes as the mass ratio increases. For instance, when PGA=0.3g and $\mu_{\text{d}}=0.35$ the average seismic reduction coefficients for EDS-I and EDS-II under both with and without forward-directivity effects are ${\bar{k}}_{\text{FD}}^{\text{I}}=29.1\;$, ${\bar{k}}_{\text{NFD}}^{\text{I}}=29.8\;$ and ${\bar{k}}_{\text{FD}}^{\text{II}}=37.6\;$, ${\bar{k}}_{\text{NFD}}^{\text{II}}=39.2\;$ for displacement, which results in reductions of 2.3% and 4.08%, respectively. When PGA=0.3g and $\mu_{\text{d}}=0.65$ the average reduction coefficients are ${\bar{k}}_{\text{FD}}^{\text{I}}=65\;$, ${\bar{k}}_{\text{NFD}}^{\text{I}}=65.2\;$ and ${\bar{k}}_{\text{FD}}^{\text{II}}=57.4\;$, ${\bar{k}}_{\text{NFD}}^{\text{II}}=60\;$ for EDS-I and EDS-II, which leads to reductions of 0.31% and 4.33%, respectively.

To investigate forward-directivity effects’ influence on the damper’s damping performance further, we define the forward-directivity influence coefficient, $f_{\text{FD}}^{\text{EDS}}$, which is based upon the average damping coefficient. The mathematical expression is as follows:


$$f_{\text{FD}}^{\text{EDS}}=\frac{{\bar{k}}_{\text{FD}}}{{\bar{k}}_{\text{NFD}}}$$ (10)

In the equation, $f_{\text{FD}}^{\text{EDS}}$ is the forward-directivity influence coefficient, in which EDS represents the Energy Dissipated Structure, FD denotes forward-directivity effects, and NFD indicates non-forward-directivity effects; ${\bar{k}}_{\text{FD}}$ is the structure’s average damping coefficient under forward-directivity effects, and ${\bar{k}}_{\text{NFD}}$ is the structure’s average damping coefficient under non-forward-directivity effects.

The forward-directivity influence coefficient, $f_{\text{FD}}^{\text{EDS}}$, for the structure’s maximum displacement, maximum acceleration, and maximum inter-story drift ratio under design earthquake conditions (PGA = 0.30g) and different mass ratios ($\mu_{\text{d}}=0.05,0.15,0.25,0.35,0.45,0.55,0.65$) are presented in Table 3.

**Table 3:** Forward-directivity influence coefficients.

		$\mu_{\text{d}}=0.05$	$\mu_{\text{d}}=0.15$	$\mu_{\text{d}}=0.25$	$\mu_{\text{d}}=0.35$	$\mu_{\text{d}}=0.45$	$\mu_{\text{d}}=0.55$	$\mu_{\text{d}}=0.65$
PGA=0.30g	**Maximum Displacement**	0.984 [0.945]	0.940 [0.999]	0.943 [0.952]	0.977 [0.958]	0.991 [0.952]	0.994 [0.957]	0.997 [0.964]
	**Maximum Acceleration**	0.851 [0.849]	0.868 [0.853]	0.912 [0.861]	0.941 [0.864]	0.961 [0.904]	0.976 [0.941]	1.006 [1.150]
	**Maximum Inter-Story Drift Ratio**	0.844 [0.831]	0.823 [0.927]	0.878 [0.991]	0.933 [0.995]	0.965 [0.997]	0.975 [1.003]	0.981 [1.011]

The values * and [*] represent the forward-directivity influence coefficients of VD and TVMD, respectively.

Table 3 shows that under the design earthquake conditions (PGA = 0.30g), the forward-directivity influence coefficients for VD and TVMD’s displacement reduction effects range from 0.940 to 1 and 0.945 to 1, with mean values of 0.975 and 0.961, respectively. The forward-directivity influence coefficients for VD and TVMD’s acceleration reduction effects range from 0.851 to 1.006 and 0.849 to 1.150, with mean values of 0.931 and 0.917, respectively. The forward-directivity influence coefficients for VD and TVMD’s inter-story drift ratio reduction effects range from 0.823 to 0.981 and 0.831 to 1.011, with mean values of 0.914 and 0.965, respectively.

These findings indicate that near-fault pulse-type ground motion records’ forward-directivity effects can diminish dampers’ effectiveness in dissipating energy, with a more pronounced effect on the acceleration reduction effect compared to the displacement and inter-story drift ratio reduction effects. This phenomenon can be attributed to the concentration of energy in specific directions because of forward-directivity effects, which results in increased local structural acceleration. In such scenarios, the dampers may not be able to respond and absorb the concentrated energy effectively, which weakens their damping performance overall thereby.

### 5.3. Influence of PGA/PGV on the control performance of TVMD

Based upon the varying values of the spectral coefficient, $f_{\text{g}}$ the 288 near-fault pulse-type ground motion records are categorized into three groups: $\begin{array}{c} f_{\text{g}}\in \text{[0},\text{10]} \end{array}$ (a total of 185 records), and $\begin{array}{c} f_{\text{g}}\in \text{[10},\text{20]} \end{array}$(a total of 82 records), and $\begin{array}{c} f_{\text{g}}\in \text{[20},\text{37]} \end{array}$ (a total of 21 records). The spectral coefficient $f_{\text{g}}$’s influence on the dampers’ damping control performance under each group of near-fault ground motion records will be discussed separately.

**Fig 11:**
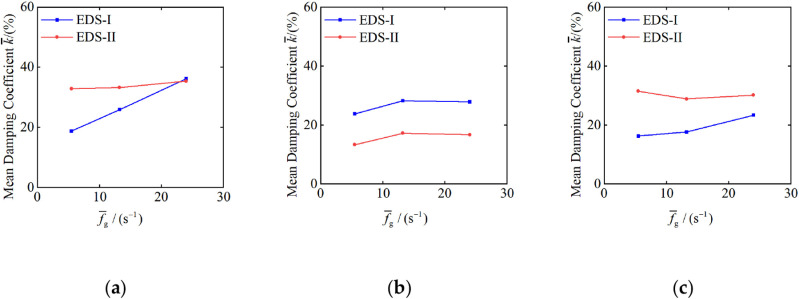
Relation between mean damping coefficient and mean spectral coefficient: (a) maximum inter-story drift ratio; (b) maximum acceleration; (c) maximum displacement.

Under the design earthquake conditions (PGA=0.30g) and a mass ratio $\mu_{\text{d}}=0.25$ the mean damping coefficients for the maximum inter-story drift ratio, maximum acceleration, and maximum displacement for both VD and TVMD were analyzed in relation to the mean spectral coefficient $f_{\text{g}}$, as illustrated in Fig 11.

As Fig 11 shows, the slopes of ${\bar{f}}_{\text{g}}$ versus the mean damping coefficient, $\bar{k}$, curves for VD are 0.94, 0.21, and 0.39 for the maximum inter-story drift ratio, maximum acceleration, and maximum displacement, respectively. In contrast, the slopes for TVMD are 0.14, 0.17, and 0.06 for the same parameters. This indicates that the slope of ${\bar{f}}_{\text{g}}$ versus $\bar{k}$ curve for VD is significantly greater than that for TVMD, suggesting that VD is more sensitive to changes in ${\bar{f}}_{\text{g}}$ and demonstrates a more pronounced damping effect on high-frequency ground motions.

## 6. Conclusions

Through dynamic time history analysis, this study investigates near-fault pulse-type ground motion characteristics’ influence on seismic structures’ maximum response distribution and TVMD and VD’s performance under different seismic conditions. The findings indicate that:

(1) Under near-fault pulse-type ground motions, when $T_{1}/T_{\text{p}}(2$ and $\mu_{\text{d}}(\text{0.45}$ as well as $T_{1}/T_{\text{p}}(2$ and $\mu_{\text{d}}(\text{0.15}$, TVMD demonstrates the dual effect of reducing energy input and increasing energy dissipation’s efficiency, showcasing inertia’s “damping enhancement” effect. However, TVMD does not achieve “damping enhancement” for the damping units. In comparison, VD exhibits greater advantages in controlling maximum acceleration, and this advantage becomes more pronounced as the mass ratio increases.

(2) When $T_{1}/T_{\text{p}}\;\leq 0.5$, pulse-type ground motion’s effects are largely lower frequency responses, and the maximum inter-story drift ratio occurs predominantly in the seismic structure’s lower levels. When $0.5<T_{1}/T_{\text{p}}\;<2$, the ground motion’s pulse effects resonate with the structure’s natural frequencies and lead to a more uniform distribution of maximum inter-story drift ratios throughout the structure’s height. When $T_{1}/T_{\text{p}}\;\geq 2$, pulse-type ground motion’s effects are concentrated at higher frequency responses that affect the seismic structure’s upper levels primarily.

(3) Near-fault pulse-type ground motion’s forward-directivity effect amplifies seismic structures’ response significantly. In addition, under forward-directivity effects, the probability that the maximum inter-story drift ratio will occur in the structure’s lower levels increases, which necessitates more attention to those levels. The forward-directivity effect reduces the dampers’ damping effect, which has a greater influence on reducing acceleration compared to displacement and reducing the inter-story drift ratio.

(4) The ground motion’s frequency spectrum coefficient, $f_{\text{g}}$, influences the distribution of the maximum acceleration response in seismic structures significantly. As $f_{\text{g}}$ increases, the location of the maximum acceleration response shifts downward along the height of the structure. Ground motions with smaller $f_{\text{g}}$ induce larger inter-story drift ratio responses in the building structure. Compared to TVMD, VD is more sensitive to changes in $f_{\text{g}}$, and demonstrates a more significant damping effect on high-frequency ground motions.

### Research prospects

The impact of different arrangements of TVMD on the structural vibration control performance and its effectiveness for various types of structures requires further investigation. Additionally, future studies should focus on the design and implementation of shaking table tests to evaluate the dynamic response of TVMD under actual seismic excitations.

## Supporting information

S1 Table Near-fault pulse-type ground motion records. (DOCX)

## References

[1] Araz O, Kahya V. Optimization of non-traditional tuned mass damper for damped structures under harmonic excitation. Uludağ University J The Faculty of Engineering. 2021;:1021–34. doi: 10.17482/uumfd.878114

[2] Wang L, Shi W, Zhou Y. Adaptive-passive tuned mass damper for structural aseismic protection including soil–structure interaction. Soil Dynamics and Earthquake Engineering. 2022;158:107298. doi: 10.1016/j.soildyn.2022.107298

[3] ARAZ O. Optimum Three-Element Tuned Mass Damper for Damped Main Structures under Ground Acceleration. El-Cezeri Fen ve Mühendislik Dergisi. 2021. doi: 10.31202/ecjse.913901

[4] ARAZ O. Optimum passive tuned mass damper systems for main structures under harmonic excitation. Mühendislik Bilimleri ve Tasarım Dergisi. 2021;9(4):1062–71. doi: 10.21923/jesd.888880

[5] Kaveh A, Fahimi Farzam M, Hojat Jalali H. Statistical seismic performance assessment of tuned mass damper inerter. Struct Control Health Monit. 2020;27(10). doi: 10.1002/stc.2602

[6] Yang F, Sedaghati R, Esmailzadeh E. Vibration suppression of structures using tuned mass damper technology: A state-of-the-art review. Journal of Vibration and Control. 2021;28(7–8):812–36. doi: 10.1177/1077546320984305

[7] Kang X, Huang Q, Wu Z, Tang J, Jiang X, Lei S. A Review of the Tuned Mass Damper Inerter (TMDI) in Energy Harvesting and Vibration Control: Designs, Analysis and Applications. CMES. 2024;139(3):2361–98. doi: 10.32604/cmes.2023.043936

[8] Saito K, Inoue N. A study on optimum response control of passive control systems using viscous damper with inertial mass Substituting equivalent nonlinear viscous elements for linear viscous elements in optimum control systems. Nihon Kenchiku Gakkai Gijutsu Hokokushu. 2007;13(26):457–62. doi: 10.3130/aijt.13.457

[9] Ikago K, Saito K, Inoue N. Seismic control of single‐degree‐of‐freedom structure using tuned viscous mass damper. Earthq Engng Struct Dyn. 2012;41(3):453–74. doi: 10.1002/eqe.1138

[10] Arai T, Aburakawa T, Ikago K, Hori N, Inoue N. Verification on effectiveness of a tuned viscous mass damper and its applicability to non-linear structural systems. Nihon Kenchiku Gakkai Kozokei Ronbunshu. 2009;74(645):1993–2002. doi: 10.3130/aijs.74.1993

[11] Smith MC. Synthesis of mechanical networks: the inerter. IEEE Trans Automat Contr. 2002;47(10):1648–62. doi: 10.1109/tac.2002.803532

[12] Ma R, Bi K, Hao H. Inerter-based structural vibration control: A state-of-the-art review. Engineering Structures. 2021;243:112655. doi: 10.1016/j.engstruct.2021.112655

[13] Chen H, Tan P. Optimal design of TVMD with linear and nonlinear viscous damping subjected to white‐noise excitation. Struct Control Health Monit. 2020;28(4). doi: 10.1002/stc.2694

[14] Cheng Y, Ji X, Ikago K, Luo H. Analytical solutions of H 2 control and efficiency‐based design of structural systems equipped with a tuned viscous mass damper. Structural Contr & Hlth. 2022;29(5). doi: 10.1002/stc.2932

[15] He H, Tan P, Hao L, Xu K, Xiang Y. Optimal design of tuned viscous mass damper for acceleration response control of civil structures under seismic excitations. Engineering Structures. 2022;252:113685. doi: 10.1016/j.engstruct.2021.113685

[16] Zhou FY, Lü YH, Zhang RF. et al. Design method of structure using inerter system based on dual control of displacement and acceleration and damping enhancement. J Build Structures. 2024;45(01):85–96. doi: 10.14006/j.jzjgxb.2022.0724

[17] He H, Tan P, Hao L, Xu K, Xiang Y. Optimal design of tuned viscous mass dampers based on effective damping ratio enhancement effect. J Sound Vibration. 2022;534:117018. doi: 10.1016/j.jsv.2022.117018

[18] Long Z, Shen W, Zhu H. On energy dissipation or harvesting of tuned viscous mass dampers for SDOF structures under seismic excitations. Mechanical Systems Signal Processing. 2023;189:110087. doi: 10.1016/j.ymssp.2022.110087

[19] Pan C, Zhang R. Design of structure with inerter system based on stochastic response mitigation ratio. Struct Control Health Monit. 2018;25(6):e2169. doi: 10.1002/stc.2169

[20] Pan C, Zhang R, Luo H, Li C, Shen H. Demand-based optimal design of oscillator with parallel-layout viscous inerter damper. Struct Control Health Monit. 2017;25(1):e2051. doi: 10.1002/stc.2051

[21] Wen Y, Chen Z, Hua X. Design and Evaluation of Tuned Inerter-Based Dampers for the Seismic Control of MDOF Structures. J Struct Eng. 2017;143(4). doi: 10.1061/(asce)st.1943-541x.0001680

[22] Ikago K, Saito K, Inoue N. Seismic control of buildings using tuned viscous mass dampers. In: Proceedings of JSSI 15th anniversary international symposium on seismic response controlled buildings for sustainable society. 2009.

[23] Ikago K, Sugimura Y, Saito K, Inoue N. Modal Response Characteristics of a Multiple-Degree-Of-Freedom Structure Incorporated with Tuned Viscous Mass Dampers. J Asian Architecture and Building Engineering. 2012;11(2):375–82. doi: 10.3130/jaabe.11.375

[24] Ji X, Cheng Y, Molina Hutt C. Seismic response of a tuned viscous mass damper (TVMD) coupled wall system. Engineering Structures. 2020;225:111252. doi: 10.1016/j.engstruct.2020.111252

[25] Ji X, Zhang J, Ikago K, Chakraborty S, Kanno H. Tuned viscous mass damper (TVMD) coupled wall system for enhancing seismic performance of high-rise buildings. Engineering Structures. 2021;240:112307. doi: 10.1016/j.engstruct.2021.112307

[26] Zhang R, Wu M, Ren X, Pan C. Seismic response reduction of elastoplastic structures with inerter systems. Engineering Structures. 2021;230:111661. doi: 10.1016/j.engstruct.2020.111661

[27] Garrido H, Curadelli O, Ambrosini D. Improvement of tuned mass damper by using rotational inertia through tuned viscous mass damper. Engineering Structures. 2013;56:2149–53. doi: 10.1016/j.engstruct.2013.08.044

[28] Hu Y, Wang J, Chen MZQ, Li Z, Sun Y. Load mitigation for a barge-type floating offshore wind turbine via inerter-based passive structural control. Engineering Structures. 2018;177:198–209. doi: 10.1016/j.engstruct.2018.09.063

[29] Zhang R, Zhao Z, Dai K. Seismic response mitigation of a wind turbine tower using a tuned parallel inerter mass system. Engineering Structures. 2019;180:29–39. doi: 10.1016/j.engstruct.2018.11.020

[30] Zhao Z, Chen Q, Zhang R, Ren X, Hu X. Variable friction‐tuned viscous mass damper and power‐flow‐based control. Structural Contr Hlth. 2021;29(3). doi: 10.1002/stc.2890

[31] Jia R, Ji X, Cheng Y, Ikago K. Seismic response control of core wall structures using tuned viscous mass damper (TVMD) outriggers. Engineering Structures. 2023;292:116546. doi: 10.1016/j.engstruct.2023.116546

[32] Chen H, Jia S, He X, et al. Dynamic characteristics of multiple inerter based dampers for suppressing harmonically forced oscillations. Struct Eng Mech. 2019;72(6):747–62.

[33] Qian F, Zuo L. Tuned nonlinear spring-inerter-damper vibration absorber for beam vibration reduction based on the exact nonlinear dynamics model. J Sound Vibration. 2021;509:116246. doi: 10.1016/j.jsv.2021.116246

[34] Kang J, Ikago K. Seismic control of multidegree‐of‐freedom structures using a concentratedly arranged tuned viscous mass damper. Earthq Engng Struct Dyn. 2023;52(14):4708–32. doi: 10.1002/eqe.3977

[35] Sugimura Y, Goto W, Tanizawa H. Response control effect of steel building structure using tuned viscous mass damper. In: Proceedings of the 15th World Conference on Earthquake Engineering. 2012. 24–8.

[36] Psycharis IN, Fragiadakis M, Stefanou I. Seismic reliability assessment of classical columns subjected to near‐fault ground motions. Earthq Engng Struct Dyn. 2013;42(14):2061–79. doi: 10.1002/eqe.2312

[37] Wang G, Zhang S, Wang C, Yu M. Seismic performance evaluation of dam-reservoir-foundation systems to near-fault ground motions. Nat Hazards. 2014;72(2):651–74. doi: 10.1007/s11069-013-1028-9

[38] Zhou J, Sun C, Dai X, Chen G. Seismic Reliability Assessment of Inelastic SDOF Systems Subjected to Near-Fault Ground Motions Considering Pulse Occurrence. Structural Durability Health Monitoring. 2019;13(4):361–78. doi: 10.32604/sdhm.2019.05171

[39] Chen G, Liu Y, Beer M. Identification of near-fault multi-pulse ground motion. Applied Mathematical Modelling. 2023;117:609–24. doi: 10.1016/j.apm.2023.01.002

[40] TAKAHASHI K, ABE M, FUJINO T. Runaway characteristics of gantry cranes for container handling by wind gust. Mechanical Engineering Journal. 2016;3(2):15-00679-15–00679. doi: 10.1299/mej.15-00679

[41] Takeuchi T, Maeda J. Unsteady wind force on an elliptic cylinder subjected to a short-rise-time gust from steady flow. J Wind Engineering and Industrial Aerodynamics. 2013;122:138–45. doi: 10.1016/j.jweia.2013.06.008

[42] Su N, Peng S, Hong N. Stochastic dynamic transient gusty wind effect on the sliding and overturning of quayside container cranes. Structure Infrastructure Engineering. 2020;17(9):1271–83. doi: 10.1080/15732479.2020.1809465

[43] Su N, Peng ST, Hong NN. Impulsive vibration response control of tuned viscous mass damper based on pole assignment approach. J Build Struct. 2023;44(09):115–23.

[44] Kang Y, Peng L, Liu Q, et al. Seismic control of tuned dampers for seismic isolated structures under near-fault pulse-like ground motions. Eng Mech. 2024;:1–14. doi: 10.6052/j.issn.1000-4750.2023.01.0034

[45] Zhao Z, Hu X, Zhang R, Chen Q. Analytical optimization of the tuned viscous mass damper under impulsive excitations. Int J Mechanical Sciences. 2022;228:107472. doi: 10.1016/j.ijmecsci.2022.107472

[46] Su N, Bian J, Peng S, Xia Y. Impulsive resistant optimization design of tuned viscous mass damper (TVMD) based on stability maximization. Int J Mechanical Sciences. 2023;239:107876. doi: 10.1016/j.ijmecsci.2022.107876

[47] Zhang L, Han J, Li D, et al. Fragility analysis of structures with tuned viscous mass dampers under near-fault pulse-type ground motions. Eng Mech. 2024;41(06):212–23.

[48] Rahman M, Nahar T, Kim D. Effect of Frequency Characteristics of Ground Motion on Response of Tuned Mass Damper Controlled Inelastic Concrete Frame. Buildings. 2021;11(2):74. doi: 10.3390/buildings11020074

[49] Araz O. Effect of PGV/PGA ratio on seismic-induced vibrations of structures equipped with parallel tuned mass dampers considering SSI. Structures. 2024;68:107188. doi: 10.1016/j.istruc.2024.107188

[50] Araz O, Elias S. Performance of differently arranged double-tuned mass dampers for structural seismic response control including soil-structure interaction. Engineering Structures. 2024;319:118841. doi: 10.1016/j.engstruct.2024.118841

[51] Djerouni S, Ounis A, Elias S, Abdeddaim M, Rupakhety R. Optimization and performance assessment of tuned mass damper inerter systems for control of buildings subjected to pulse-like ground motions. Structures. 2022;38:139–56. doi: 10.1016/j.istruc.2022.02.007

[52] Elias S, Djerouni S. Optimum tuned mass damper inerter under near-fault pulse-like ground motions of buildings including soil-structure interaction. J Building Engineering. 2024;85:108674. doi: 10.1016/j.jobe.2024.108674

[53] Housner GW, Hudson DE. The Port Hueneme earthquake of March 18, 1957. Bulletin of the Seismological Society America. 1958;48(2):163–8. doi: 10.1785/bssa0480020163

[54] Sehhati R, Rodriguez-Marek A, ElGawady M, Cofer WF. Effects of near-fault ground motions and equivalent pulses on multi-story structures. Engineering Structures. 2011;33(3):767–79. doi: 10.1016/j.engstruct.2010.11.032

[55] Chioccarelli E, Iervolino I. Near‐source seismic hazard and design scenarios. Earthq Engng Struct Dyn. 2012;42(4):603–22. doi: 10.1002/eqe.2232

[56] Hayden CP, Bray JD, Abrahamson NA. Selection of Near-Fault Pulse Motions. J Geotech Geoenviron Eng. 2014;140(7). doi: 10.1061/(asce)gt.1943-5606.0001129

[57] Eftekhari N, Kowsari M, Sayyadpour H. An Artificial Neural Network for Predicting the Near-fault Directivity-pulse Period. J Earthquake Engineering. 2021;26(9):4681–700. doi: 10.1080/13632469.2020.1838358

[58] Tothong P, Cornell CA. Structural performance assessment under near‐source pulse‐like ground motions using advanced ground motion intensity measures. Earthq Engng Struct Dyn. 2008;37(7):1013–37. doi: 10.1002/eqe.792

[59] Chen H, Tan P. Optimal design of TVMD with linear and nonlinear viscous damping subjected to white‐noise excitation. Struct Control Health Monit. 2020;28(4). doi: 10.1002/stc.2694

[60] Zhao Z, Hu X, Zhang R, Chen Q. Analytical optimization of the tuned viscous mass damper under impulsive excitations. Int J Mechanical Sci. 2022;228:107472. doi: 10.1016/j.ijmecsci.2022.107472

