# Supplementary material for: Influence of near-fault ground motions’ characteristics on the control performance of tuned viscous mass damper systems
Source: PLoS One. 2025 Jun 3;20(6):e0322535. doi: 10.1371/journal.pone.0322535 (PMC12133015; doi:10.1371/journal.pone.0322535)
Supplement: S1 Table — (DOCX) [file pone.0322535.s001.docx]

Appendix

**Near-fault pulse-type ground motion records**

| **NGA number^a^** | **Earthquake** | **Station** | ***M*_w_** | **Closest**  **Distance/(km)** | **Pulse period(s)** |
| --- | --- | --- | --- | --- | --- |
| 4101 | Parkfield-02, CA | Parkfield-Cholame 3E | 6 | 5.6 | 0.5 |
| 4126 | Parkfield-02, CA | Parkfield-Stone Corral 1E | 6 | 3.8 | 0.5 |
| 4482 | L’Aquila, Italy | L’Aquila-V. Aterno-F.Aterno | 6.3 | 6.6 | 0.5 |
| 461 | Morgan Hill | Halls Valley | 6.2 | 3.5 | 0.6 |
| 496 | Nahanni, Canada | Site 2 | 6.8 | 4.9 | 0.6 |
| 3966 | Tottori, Japan | TTR009 | 6.6 | 8.8 | 0.6 |
| **4103** | Parkfield-02, CA | Parkfield-Cholame 4W | 6 | 4.2 | 0.6 |
| 4104 | Parkfield-02, CA | Parkfield-Cholame 4AW | 6 | 5.5 | 0.6 |
| 828 | Cape Mendocino | Petrolia | 7 | 8.2 | 0.7 |
| **4102** | Parkfield-02, CA | Parkfield-Cholame 3W | 6 | 3.6 | 0.7 |
| 4352 | Umbria Marche, Italy | Nocera Umbria | 6 | 8.9 | 0.7 |
| 4480 | L’Aquila, Italy | L’Aquila-V. Aterno-Centro Valle | 6.3 | 6.3 | 0.7 |
| 6877 | Joshua Tree, CA | Indio-Jackson Road | 6.1 | 25.5 | 0.7 |
| **451** | Morgan Hill | Coyote Lake Dam(SWAbut) | 6.2 | 0.5 | 0.8 |
| 1004 | Northridge-01 | LA-Sepulvada VA Hospital | 6.7 | 8.4 | 0.8 |
| **1051** | Northridge-01 | Pacoima Dam(upper left) | 6.7 | 7 | 0.8 |
| 3968 | Tottori, Japan | TTRH02 | 6.6 | 1 | 0.8 |
| **4100** | Parkfield-02, CA | Parkfield-Cholame 2WA | 6 | 3 | 0.8 |
| 4116 | Parkfield-02, CA | Parkfield-Stone Corral 4E | 6 | 8.8 | 0.8 |
| **1602** | Duzce, Turkey | Bolu | 7.1 | 12 | 0.9 |
| **1752** | Northwest China-03 | Jiashi | 6.1 | 17.7 | 0.9 |
| 2627 | Chi-Chi, Taiwan-03 | TCU076 | 6.2 | 14.7 | 0.9 |
| 3475 | Chi-Chi, Taiwan-06 | TCU080 | 6.3 | 10.2 | 0.9 |
| 4065 | Parkfield-02, CA | Parkfield-Eades | 6 | 2.9 | 0.9 |
| **4099** | Parkfield-02, CA | Parkfield-Cholam 2E | 6 | 4.1 | 0.9 |
| 4481 | L’Aquila, Italy | L’Aquila-V. Aterno-Colle Grilli | 6.3 | 6.8 | 0.9 |
| **4084** | Parkfield-02, CA | Parkfield-1-Story S. | 6 | 2.7 | 1 |
| 4107 | Parkfield-02, CA | Parkfield-Fault Zone 1 | 6 | 2.5 | 1 |
| **4115** | Parkfield-02, CA | Parkfield-Fault Zone 12 | 6 | 2.7 | 1 |
| **4130** | Parkfield-02, CA | Parkfield-Vineyard Cany 1E | 6 | 3 | 1 |
| **529** | N.Palm Springs | North Palm Springs Post Office | 6.1 | 4 | 1.1 |
| 1063 | Northridge-01 | Rinaldi Receiving Sta | 6.7 | 6.5 | 1.1 |
| **3548** | Loma Prieta | Los Gatos-Lexington Dam | 6.9 | 5 | 1.1 |
| **4098** | Parkfield-02, CA | Parkfield-Cholame 1E | 6 | 3 | 1.1 |
| 4113 | Parkfield-02, CA | Parkfield-Fault Zone 9 | 6 | 2.9 | 1.1 |
| 4879 | Chuetsu-oki | Yan Sakuramachi City | 6.8 | 19 | 1.2 |
| 285 | Irpinia, Italy-01 | Bagnoli Irpinio | 6.9 | 8.2 | 1.3 |
| 763 | Loma Prieta | Gilroy-Gavilan Coll. | 6.9 | 10 | 1.4 |
| 765 | Loma Prieta | Gilroy Array #1 | 6.9 | 9.6 | 1.4 |
| **1119** | Kobe, Japan | Takarazuka | 6.9 | 0.3 | 1.4 |
| 2618 | Chi-Chi, Taiwan-03 | TCU065 | 6.2 | 26.1 | 1.4 |
| 3943 | Tottori, Japan | SMN015 | 6.6 | 9.1 | 1.4 |
| 764 | Loma Prieta | Gilroy-Historic Bldg. | 6.9 | 11 | 1.5 |
| 766 | Loma Prieta | Gilroy Array #2 | 6.9 | 11.1 | 1.5 |
| **4040** | Bam, Iran | Bam | 6.6 | 1.7 | 1.6 |
| 4228 | Niigata, Japan | NIGH11 | 6.6 | 8.9 | 1.6 |
| 4451 | Montenegro, Yugo. | Bar-Skupstina Opstine | 7.1 | 7 | 1.6 |
| 4458 | Montenegro, Yugo. | Ulcinj-Hotel Olimpic | 7.1 | 5.8 | 1.6 |
| 3746 | Cape Mendocino | Centerville Beach, Naval Fac | 7 | 18.3 | 1.7 |
| 517 | N.Palm Springs | Desert Hot Springs | 6.1 | 6.8 | 1.8 |
| 159 | Imperial Valley-06 | Agrarias | 6.5 | 0.7 | 1.9 |
| **723** | Superstition Hills-02 | Parachute Test Site | 6.5 | 1 | 1.9 |
| 4483 | L’Aquila, Italy | L’Aquila-Parking | 6.3 | 5.4 | 1.9 |
| 4847 | Chuetsu-oki | Joetsu Kakizakiku kakizaki | 6.8 | 11.9 | 1.9 |
| 767 | Loma Prieta | Gilroy Array #3 | 6.9 | 12.8 | 2 |
| 722 | Superstition Hills-02 | Kornbloom Road(temp) | 6.5 | 18.5 | 2.1 |
| **1013** | Northridge-01 | LA Dam | 6.7 | 5.9 | 2.3 |
| **2114** | Denali, Alaska | TAPS Pump Station #10 | 7.9 | 2.7 | 2.3 |
| 1077 | Northridge-01 | Santa Monica City Hall | 6.7 | 26.5 | 2.4 |
| 6906 | Darfield, NZ | GDLC | 7 | 1.2 | 2.4 |
| 821 | Erzican, Turtey | Erzincan | 6.7 | 4.4 | 2.5 |
| **1086** | Northridge-01 | Sylmar-Olive View Med FF | 6.7 | 5.3 | 2.5 |
| **1529** | Chi-Chi, Taiwan | TCU102 | 7.6 | 1.5 | 2.6 |
| 725 | Superstition Hills-02 | Poe Road(temp) | 6.5 | 11.2 | 2.7 |
| **1084** | Northridge-01 | Sylmar-Converter Sta | 6.7 | 5.4 | 2.8 |
| **1045** | Northridge-01 | Newhall-W Pico Canyon Rd. | 6.7 | 5.5 | 2.9 |
| 8130 | Christchurch, New Zealand | SHLC | 6.2 | 5.6 | 2.9 |
| **171** | Imperial Valley-06 | EI Centro-Meloland Geot.Arry | 6.5 | 0.1 | 3 |
| **1085** | Northridge-01 | Sylmar-Converter Sta East | 6.7 | 5.2 | 3 |
| 3947 | Tottori, Japan | SMNH01 | 6.6 | 5.9 | 3 |
| 292 | Irpinia, Italy-01 | Sturno(STN) | 6.9 | 10.8 | 3.1 |
| 5658 | Iwate | IWTH26 | 6.9 | 6 | 3.1 |
| **1050** | Northridge-01 | Pacoima Dam(downstr) | 6.7 | 7 | 3.2 |
| **779** | Loma Prieta | LGPC | 6.9 | 3.9 | 3.3 |
| 8123 | Christchurch, New Zealand | REHS | 6.2 | 5.1 | 3.3 |
| 8158 | Christchurch, New Zealand | LPCC | 6.2 | 6.1 | 3.4 |
| 2650 | Chi-Chi, Taiwan-03 | TCU116 | 6.2 | 22.1 | 3.5 |
| **181** | Imperial Valley-06 | EI Centro Arry #6 | 6.5 | 1.4 | 3.6 |
| **182** | Imperial Valley-06 | EI Centro Arry #7 | 6.5 | 0.6 | 3.6 |
| **1158** | Kocaeli, Turkey | Duzce | 7.5 | 15.4 | 3.9 |
| **4071** | Parkfield-02, CA | Parkfield-Middle Mtn | 6 | 2.6 | 3.9 |
| 1165 | Kocaeli, Turkey | Izmit | 7.5 | 7.2 | 4 |
| 2628 | Chi-Chi, Taiwan-03 | TCU078 | 6.2 | 7.6 | 4 |
| **180** | Imperial Valley-06 | EI Centro Arry #5 | 6.5 | 4 | 4.2 |
| 1193 | Chi-Chi, Taiwan | CHY024 | 7.6 | 9.6 | 4.2 |
| 825 | Cape Mendocino | Cape Mendocino | 7 | 7 | 4.4 |
| **879** | Landers | Lucerne | 7.3 | 2.2 | 4.4 |
| **1176** | Kocaeli, Turkey | Yarimca | 7.5 | 4.8 | 4.4 |
| **1511** | Chi-Chi, Taiwan | TCU076 | 7.6 | 2.7 | 4.5 |
| **179** | Imperial Valley-06 | EI Centro Arry #4 | 6.5 | 7.1 | 4.6 |
| 286 | Irpinia, Italy-01 | Bisaccia | 6.9 | 21.3 | 4.6 |
| **1476** | Chi-Chi, Taiwan | TCU029 | 7.6 | 28 | 4.6 |
| 8119 | Christchurch, New Zealand | PRPC | 6.2 | 2 | 4.6 |
| 1244 | Chi-Chi, Taiwan | CHY101 | 7.6 | 9.9 | 4.7 |
| **1510** | Chi-Chi, Taiwan | TCU075 | 7.6 | 0.9 | 4.7 |
| **161** | Imperial Valley-06 | Brawley Airport | 6.5 | 10.4 | 4.8 |
| **3473** | Chi-Chi, Taiwan-06 | TCU078 | 6.3 | 11.5 | 4.8 |
| **1548** | Chi-Chi, Taiwan | TCU128 | 7.6 | 13.1 | 4.9 |
| 3744 | Cape Mendocino | Bunker Hill FAA | 7 | 12.2 | 4.9 |
| 143 | Tabas, Iran | Tabas | 7.4 | 2.1 | 5.3 |
| **1480** | Chi-Chi, Taiwan | TCU036 | 7.6 | 19.8 | 5.4 |
| 3852 | Chi-Chi(after  shock3) | CHY006 | 6.2 | 24.6 | 5.4 |
| **1483** | Chi-Chi, Taiwan | TCU040 | 7.6 | 22.1 | 5.7 |
| 2658 | Chi-Chi, Taiwan-03 | TCU129 | 6.2 | 12.8 | 5.7 |
| **77** | San Fernando | Pacoima Dam | 6.6 | 1.8 | 5.9 |
| **1481** | Chi-Chi, Taiwan | TCU038 | 7.6 | 25.4 | 5.9 |
| **183** | Imperial Valley-06 | EI Centro Arry #8 | 6.5 | 3.9 | 6.1 |
| **802** | Loma Prieta | Saratoga-Aloha Ave | 6.9 | 8.5 | 6.2 |
| **1549** | Chi-Chi, Taiwan | TCU129 | 7.6 | 1.8 | 6.2 |
| **6962** | Darfield, New Zealand | ROLC | 7 | 1.5 | 6.2 |
| **803** | Chi-Chi, Taiwan | Saratoga-W Valley Coll | 6.9 | 9.3 | 6.3 |
| **6927** | Darfield, New Zealand | LINC | 7 | 7.1 | 6.3 |
| **1501** | Chi-Chi, Taiwan | TCU063 | 7.6 | 9.8 | 6.4 |
| **173** | Imperial Valley-06 | EI Centro Arry #10 | 6.5 | 8.6 | 6.7 |
| 6897 | Darfield, New Zealand | DSLC | 7 | 8.5 | 6.7 |
| **1486** | Chi-Chi, Taiwan | TCU046 | 7.6 | 16.7 | 6.8 |
| **1148** | Kocaeli, Turkey | Arcelik | 7.5 | 13.5 | 6.9 |
| 6911 | Darfield, New Zealand | HORC | 7 | 7.3 | 7.1 |
| 8090 | Christchurch, New Zealand | HPSC | 6.2 | 4.4 | 7.1 |
| **1530** | Chi-Chi, Taiwan | TCU103 | 7.6 | 6.1 | 7.4 |
| **184** | Imperial Valley-06 | EI Centro Differential Arry | 6.5 | 5.1 | 7.5 |
| 185 | Imperial Valley-06 | Holtville Post Office | 6.5 | 7.5 | 7.5 |
| 2661 | Chi-Chi, Taiwan-03 | TCU138 | 6.2 | 22.1 | 7.5 |
| **6887** | Darfield, New Zealand | CBGS | 7 | 18.1 | 7.7 |
| **6960** | Darfield, New Zealand | RHSC | 7 | 13.6 | 7.7 |
| **6959** | Darfield, New Zealand | REHS | 7 | 19.5 | 7.8 |
| **6928** | Darfield, New Zealand | LPCC | 7 | 25.7 | 8 |
| **900** | Landers | Yermo Fire Station | 7.3 | 23.6 | 8.1 |
| **1496** | Chi-Chi, Taiwan | TCU056 | 7.6 | 10.5 | 8.2 |
| **6890** | Darfield, New Zealand | CMHS | 7 | 17.6 | 8.4 |
| **1550** | Chi-Chi, Taiwan | TCU136 | 7.6 | 8.3 | 8.5 |
| **1528** | Chi-Chi, Taiwan | TCU101 | 7.6 | 2.1 | 8.6 |
| **6966** | Darfield, New Zealand | SHLC | 7 | 22.3 | 8.6 |
| 178 | Imperial Valley-06 | EI Centro Arry #3 | 6.5 | 12.9 | 8.7 |
| **6942** | Darfield, New Zealand | NNBS | 7 | 26.8 | 8.9 |
| **1519** | Chi-Chi, Taiwan | TCU087 | 7.6 | 7 | 9 |
| **1485** | Chi-Chi, Taiwan | TCU045 | 7.6 | 26 | 9.8 |
| **170** | Imperial Valley-06 | EC County Center FF | 6.5 | 7.3 | 10^b^ |
| **1491** | Chi-Chi, Taiwan | TCU051 | 7.6 | 7.6 | 10^b^ |
| **1492** | Chi-Chi, Taiwan | TCU052 | 7.6 | 0.7 | 10^b^ |
| **1494** | Chi-Chi, Taiwan | TCU054 | 7.6 | 5.3 | 10^b^ |
| **1505** | Chi-Chi, Taiwan | TCU068 | 7.6 | 0.3 | 10^b^ |
| **1515** | Chi-Chi, Taiwan | TCU082 | 7.6 | 5.2 | 10^b^ |
| 1520 | Chi-Chi, Taiwan | TCU088 | 7.6 | 18.2 | 10^b^ |

^a^Bold NGA record numbers indicate motion was classified as a FD-pulse.

^b^Pulse period 10s or greater.
